# Rickettsiales Occurrence and Co-occurrence in *Ixodes ricinus* Ticks in Natural and Urban Areas

**DOI:** 10.1007/s00248-018-1269-y

**Published:** 2018-10-16

**Authors:** Maciej Kowalec, Tomasz Szewczyk, Renata Welc-Falęciak, Edward Siński, Grzegorz Karbowiak, Anna Bajer

**Affiliations:** 1 Department of Parasitology, Institute of Zoology, Faculty of Biology, University of Warsaw, 1 Miecznikowa Street, 02-096 Warszawa, Poland; 2 W. Stefański Institute of Parasitology of the Polish Academy of Sciences, 51/55 Twarda Street, 00-818 Warszawa, Poland

**Keywords:** Rickettsiales, *Rickettsia helvetica*, *Anaplasma phagocytophilum*, Neoehrlichia mikurensis, *Ixodes ricinus*, Urban, Natural, City, Co-infection

## Abstract

Bacteria of Rickettsiaceae and Anaplasmataceae families include disease agents spread by *Ixodes ricinus* ticks, the most common tick vector in Europe. The aim of the study was to compare the prevalence and co-infection prevalence of particular tick-transmitted Rickettsiales members: *Rickettsia* spp. (further referred as Rs), *Anaplasma phagocytophilum* (Ap), and “*Candidatus* Neoehrlichia mikurensis” (CNM) in *I*. *ricinus* ticks in two types of areas, different in terms of human impact: natural and urban. Using additional data, we aimed at investigating co-occurrence of these Rickettsiales with *Borreliella* spp. A total of 4189 tick specimens, 2363 from the urban area (Warsaw park and forests) and 1826 from the natural area (forests and park in the vicinity of National Parks), were tested for the presence of Rickettsiales DNA by PCRs. The prevalence of selected Rickettsiales was twice higher in urban than natural areas (13.2% vs. 6.9%, respectively). In total ticks, the prevalence of Rs, Ap, and CNM was 6.5%, 5.3%, and 3.6% in urban areas vs. 4.4%, 1.1%, and 2.1% in natural areas, respectively. Co-infections of Rickettsiales were also more prevalent in urban areas (2.6% vs. 0.3%, respectively). The most common Rs was *R*. *helvetica*; also *R*. *monacensis* and novel “*Candidatus* Rickettsia mendelii” were detected. Positive association between Ap and CNM infections was discovered. Rickettsiales bacteria occurrence was not associated with *Borreliella* occurrence, but co-infections with these two groups were more common in ticks in urban areas. In conclusion, three groups of Rickettsiales constituted the important part of the tick pathogen community in Poland, especially in the urbanized central Poland (Mazovia). In the Warsaw agglomeration, there is a greater risk of encountering the *I*. *ricinus* tick infected with Rickettsiales and co-infected with Lyme spirochaetes, in comparison to natural areas. This finding raises the question whether cities might in fact be the hot spots for TBDs.

**Electronic supplementary material:**

The online version of this article (10.1007/s00248-018-1269-y) contains supplementary material, which is available to authorized users.

## Background

The order Rickettsiales contains tick-transmitted bacteria, causing many types of rickettsioses in both humans and animals. Particularly, the Rickettsiaceae and Anaplasmataceae families include disease agents spread by *Ixodes ricinus* ticks, the most common and significant tick vector in Europe [1–3]. The *I*. *ricinus*-borne infectious diseases are of great importance in many European countries, including few considered as emerging diseases [4].

The *Rickettsia* genus (fam. Rickettsiaceae) comprises a diverse group of vector-borne bacteria transmitted by ectoparasitic arthropods, both insects (fleas, lice) and arachnids (ticks and mites). Bacteria transmitted by ticks are known as Spotted Fever (SF) rickettsiae, since those species which are more pathogenic cause a rash and fever. The most common species found in *I*. *ricinus* in Poland is *Rickettsia helvetica*, which is considered of relatively low pathogenicity (reviewed in [5, 6]), though it may cause cardiomyopathy [7]. Because *Rickettsia* spp. are transmitted both trans-stadially and trans-ovarially in a vector population without the need for an external source of infection, ticks are known as their main reservoir [5, 8]. However, these pathogens were also detected in numerous vertebrate hosts such as birds, reptiles, and mammals [8–10].

Bacteria of the Anaplasmataceae family such as *Anaplasma phagocytophilum* and the novel pathogen “*Candidatus* Neoehrlichia mikurensis” are also transmitted by *I*. *ricinus* ticks in Europe (reviewed in [3, 11, 12]). Rodents and ruminants constitute the main reservoir of these pathogens; however, bacteria can also infect different groups of mammals or birds (reviewed in: [11, 13]). Both species cause a fever and an influenza-like illness with nonspecific symptoms, mild to severe in cases of immunodeficiency: human granulocytic anaplasmosis (HGA) and neoehrlichiosis [11, 14–16].

During the course of an infection, *A*. *phagocytophilum* cripples neutrophil-dependent mechanisms of immunological defense, which may be associated with a decrease of native immunity and an increase of susceptibility to other infections, in some cases causing a serious threat to the health of the host [14]. The co-infections of *A*. *phagocytophilum* and other tick-borne pathogens (TBPs) in ticks are not uncommon [17]. In humans, the simultaneous infection of *A*. *phagocytophilum* and Lyme spirochaetes is particularly well known, which due to immuno-regulating features of *A*. *phagocytophilum* may contribute to an increase in the severity of Lyme disease in humans [18, 19]. Additionally, of epidemiological importance is that these simultaneous infections may also facilitate the transmission of each pathogen from host to vector [20]. Co-infections of this kind were already reported in Poland [21, 22].

The biology of “*Ca*. N. mikurensis” (CNM) has not been well recognized since this species is not cultivable to date. Despite that, CNM is widely detected, distributed both in vector and tick hosts [11]. The presence of CNM was detected also in immunocompetent healthy humans [23] signalizing a potential threat for health, e.g., in the case of immunosuppression or blood transfusion to immunocompromised individuals. It is still to be investigated whether CNM may also affect transmission and the infection process of other disease agents similarly to *A*. *phagocytophilum*.

*Ixodes ricinus* ticks constitute a serious public health threat not only in forests, but also in cities—in suburban and urban forests, parks, and recreational sites [1, 24]. Numerous studies focused on the role of urban ticks as vectors of *Borreliella* spp. (new genus created for *Borrelia burgdorferi* sensu lato spirochaetes after recent division of *Borrelia* genus [25–27]), but their role as the vectors of Rickettsiales is less recognized. In Poland, tick-borne rickettsioses caused by *Rickettsia* spp. are rarely diagnosed, despite an annual no. of ~ 20,000 cases of Lyme disease, indicating an elevated risk of tick bites and transmission of TBPs [28]. It is plausible, however, that these rickettsial infections are often unnoticed or misdiagnosed—especially due to unspecific symptoms and particularly due to the fact that HGA and neoehrlichiosis are not registered diseases in Poland. In our previous report [29], we found that urbanization or high human impact on the environment may positively affect the rickettsia circulation in ticks (resulting in a more common occurrence) and therefore in cities, there may be in fact an increased risk of acquiring tick-borne rickettsioses.

The aim of the study was to compare the prevalence of particular Rickettsiales: *Rickettsia* spp. (further referred as Rs), *A*. *phagocytophilum* (Ap), and “*Ca*. N. mikurensis” (CNM) in *I*. *ricinus* ticks in two types of areas, different in terms of human impact: natural and urban. Additionally, we made an attempt to detect and analyze co-infections in ticks from these areas based on our previous study on *Borreliella* spp. infection, to evaluate if human impact on the environment may affect co-occurrence of pathogenic Rickettsiales and Lyme spirochaetes, increasing the risk of TBDs transmission and emergence.

## Methods

### Field Study: Tick Collection and Research Areas

Ticks were collected by flagging from 2012 to 2015 in two ecologically different (in terms of level of human impact) types of areas: natural and urban. The sampling was performed as described previously [24], at six selected study sites in the same locations and marked spots in spring-summer and summer-autumn seasons (study sites classification is provided in Suppl. File 1). Constant spots at each study site were flagged at least once in each season of the study. The number of ticks collected was recalculated per 100 m^2^ for each individual flagging event (sampling at specific date on designated spots).

Three low-transformed forested areas in NE and Central Poland were selected as natural areas: the Mazurian Landscape Park (MLP), Białowieża National Park (BNP), and Kampinoski National Park (KNP). The study sites in natural areas were situated in protected areas or in proximity to nature reserves.

The natural study site within the Mazurian Landscape Park (MLP; described in [30] [53°47′47″ N 21°39′49″ E] is situated in NE Poland, within the Mazurian Lake District. It is a mixed forest surrounded by lakes and re-cultivated meadows.

Kampinoski National Park (KNP) is a vast forest complex north and north-west of Warsaw. Three particular study sites were selected in the eastern part of KNP: “Dziekanów Leśny” [52°20′14.1″ N, 20°50′04.4″ E], “Palmiry” [52°19′53.8″ N, 20°44′50.1″ E], and “Truskaw” [52°18′37.2″ N, 20°45′37.6″ E], distant from each other but representing one continuous forest complex, so ticks collected in these localities were grouped as originating from one site (KPN) for analysis. Both MLP and KNP sites were described in detail previously [29].

The three urban sites involved two city forests: Bielański Forest (WBF) and Kabacki Forest (WKF); and a city park in Warsaw: Royal Łazienki Park (WLP), which could be compared with the park in Białowieża in the sense of maintenance and fencing. Thus, we were able to categorize sites further into two subtypes of each area both in urban and natural areas: forests and parks (Suppl. File 1).

The detailed description of the Białowieża (BNP) natural area and all urban sites within the Warsaw agglomeration (two forests and park) is provided in our previous publications [24, 29].

### Laboratory Study

Species and stage of ticks were identified with the use of zoological keys [31, 32]. Ticks were further subjected to PCR screening for rickettsial DNA. Genomic DNA from ticks was isolated with Genomic Tissue Spin-Up kit (AA Biotechnology, Gdynia, Poland) according to the manufacturer’s protocol, from individual specimens of adults and from pools of 10 nymphs. Genomic DNA was used for molecular screening of rickettsiae by amplification of selected molecular markers with the use of Dream Taq polymerase and DreamTaq Green Buffer (Thermo Fisher Scientific Baltics UAB, Vilnius, Lithuania). The amplicons were visualized on 1.5% agarose gel stained with Midori Green Stain (Nippon Genetics Europe, Düren, Germany).

Two different genes were selected for different aims: amplification of 16S rDNA fragments with species-specific primers enabled the specific detection of CNM and Ap, and citrate synthase (*gltA*) gene was chosen for the detection and species identification of *Rickettsia* spp., as we expected several species of *Rickettsia* in *I*. *ricinus* ticks from studied areas.

Primers CS409 and Rp1258 were used for the amplification of a 769 bp fragment of the Rs *gltA* gene [33] in modified conditions: initial denaturation in 95 °C for 5 min, 40 cycles of denaturation at a temperature of 95 °C for 30 s, 45 s of primer annealing in 59 °C, and elongation in 65 °C for 1 min. For both Ap and CNM detection, a two-step nested PCR protocol was used for the amplification of 16S rRNA gene (*rss*) fragments. In the first step of the PCR reaction, DNA of both species of bacteria was amplified with previously published, Anaplasmataceae-specific primers EC9/EC12A [34]. In the second PCR, two sets of primers were used separately: primers Ge9F/Ge10r (950 bp product) specific for Ap [35] and primers IS58-132f/IS58-654r (470 bp product) specific for CNM [34]. The reaction conditions for both Ap and CNM PCR protocols were as follows: initial denaturation in 95 °C for 5 min, 40 cycles of denaturation in 95 °C for 30 s, 30 s of primer annealing in 55 °C, and elongation in 72 °C for 1 min for the primary (outer) reaction. In the secondary (inner/nested) reaction, primer annealing was conducted in 57 °C.

For species-typing of Rs and confirmation of species-specific amplification of Ap and CNM DNAs, a representative number of PCR products were sequenced by an outsource company (Genomed, Warsaw, Poland).

### In Silico Analysis

For statistical comparisons, particular sites in Białowieża and in KNP were considered as unified BNP and KNP sites. Thus, in further analyses, three sites in natural areas and three sites in urban areas were compared (Suppl. File 1). Analysis of tick abundance was performed for KNP and MLP as described in our previous study [24].

The prevalence of tick infections (percentage of ticks infected) for each pathogen and for a combination of pathogens was calculated. Minimum infection rate (MIR) for pools of nymphs was calculated, assuming that only one specimen in a pool was positive in a positive sample. Thus, the prevalence of infection in total ticks incorporated the MIR. Percentages of Rickettsiales infections were analyzed in IBM SPSS 20.0 Software by maximum likelihood techniques based on log-linear analysis of contingency tables (HILOGLINEAR). For analysis of the prevalence of Rickettsiales (Rs, Ap, and CNM) in ticks, we fitted the *presence*/*absence* of each bacterial group as a binary factor (infected = 1, uninfected = 0) and then *Year* (4 levels: 2012–2015), *Season* (spring-summer or summer-autumn) and *Type of area* (urban or natural), or *Site* (1–6) in the second model. Additionally, a *Subtype of area* was inserted into the analysis in a different model (1 = forest, 2 = park; in this case the particular sites in Białowieża were analyzed separately). A minimum sufficient model was then obtained, for which the likelihood ratio of χ^2^ was not significant, indicating that the model was sufficient in explaining the data.

The same statistical approach was used to investigate the factors influencing the occurrence of co-infections in adult ticks. The first step was the analysis of occurrence of Rickettsiales co-infection (“Rs and Ap”; “Rs and CNM” and “Ap and CNM”). Additionally, co-infections of Rickettsiales with spirochaetes were likewise analyzed in tick samples from 2013 to 2015. Data on the prevalence of spirochaetes (*Borreliella* spp. and/or *Borrelia miyamotoi*) in ticks from the same study sites were published recently [24]. The expected co-infection rates/frequencies were calculated according to the prevalence of individual bacteria species, as the product of infection percentages. For example, if the prevalence of Ap is 3.5% and CNM is 2.9% in total ticks, then the expected prevalence of Ap and CNM co-infection in total ticks is 3.5% × 2.9% = 0.1%.

In the second step, HILOGLINEAR analysis was performed with an additional factor—the secondary infection status (non-infected = 0 or infected with second pathogen = 1) to check whether infection with one investigated pathogen could have affected the infection with another. In addition, we have compared the prevalence of one pathogen in a group of infected and non-infected with another pathogen, with the use of Fisher’s exact test to confirm the HILOGLINEAR model prediction.

Obtained sequences of Rs, Ap, and CNM were compared with GenBank-deposited sequences through the BLAST-NCBI algorithm and representative consensus sequences were deposited in the GenBank database.

## Results

Overall, 5321 ticks were collected in total of 389 recorded individual samplings by flagging: 175 from natural areas and 214 from urban areas. In addition to the data on tick abundance, presented for the Białowieża and Warsaw sites included in our previous report [24], tick abundance was calculated for KNP and MLP. *Ixodes ricinus* ticks were twice as abundant in MLP in comparison to KNP (6.6 ± 1.7 vs. 3 ± 2, respectively) (Suppl. File 2). The *Type of area* has no effect on tick abundance—no significant differences were found between natural and urban areas in this study (data not shown).

Of the 4189 ticks collected, 79% were subjected to molecular screening for Rickettsiales (Rs, Ap, and CNM) bacteria: including 2030 adults (898 from natural areas and 1132 from urban areas) and 2159 nymphs, grouped in 223 pools. In total, 10.4% (437/4189) of the ticks were positive for the presence of DNA of at least one species of Rickettsiales bacteria. Overall, 16.7% (340/2030) of adult ticks and 43.5% (97/223) of the pools of nymphs [MIR = 4.5% (97/2159)] were positive for any of the Rickettsiales DNA.

**Fig. 1:**
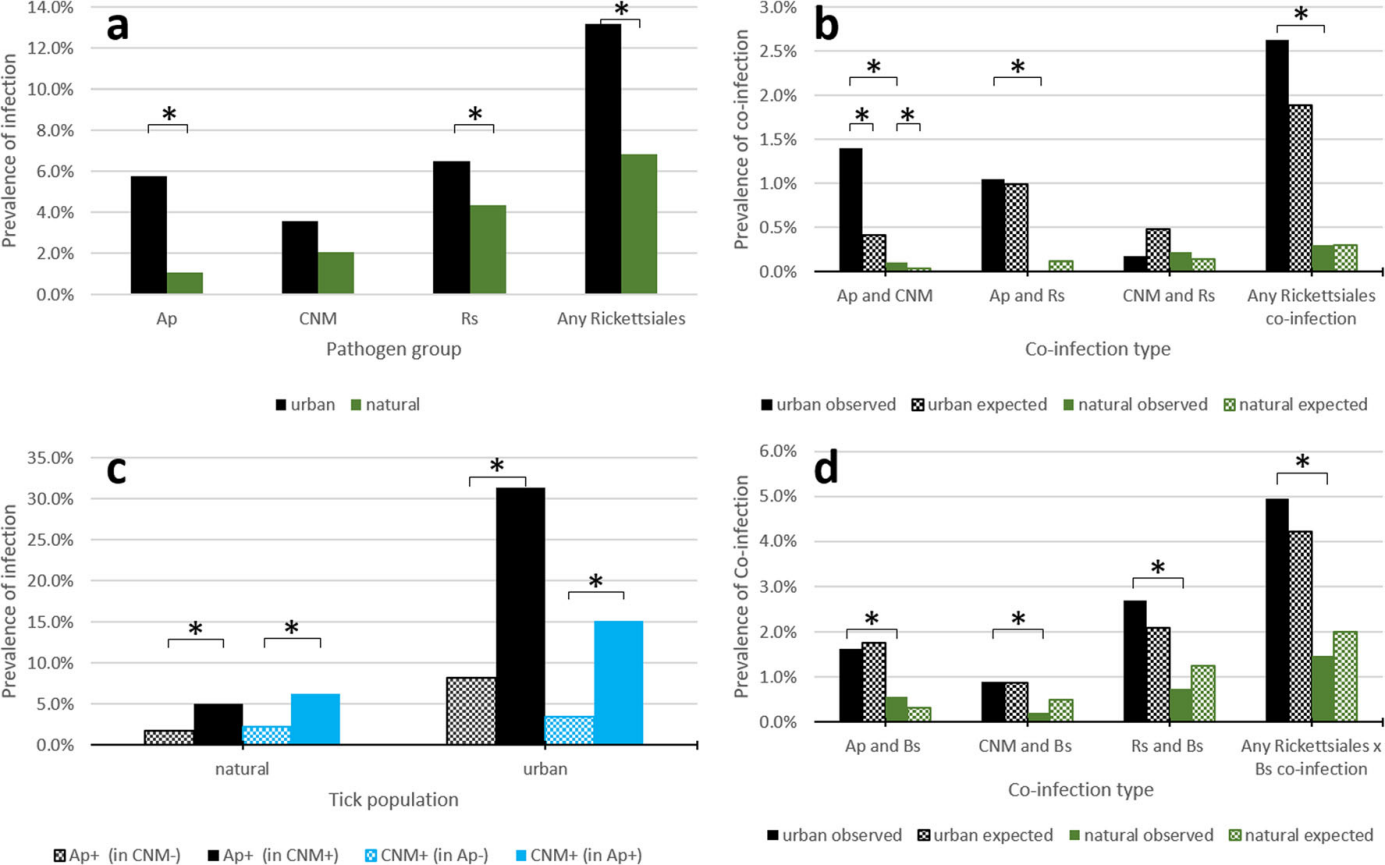
Prevalence of infections and co-infections of selected Rickettsiales in *Ixodes ricinus* ticks in urban and natural areas. **a** Prevalence of Rickettsiales in urban and natural areas. **b** Prevalence of Rickettsiales co-infections in urban and natural areas. **c** Prevalence of Ap and CNM in urban and natural areas in a group uninfected and infected with a second pathogen (CNM or Ap, respectively). **d** Prevalence of Rickettsiales and *Borreliella* spirochaetes (Bs) co-infection in urban and natural areas. Abbreviations: *Ap Anaplasma phagocytophilum*, *CNM* “*Candidatus* Neoehrlichia mikurensis,” *Rs Rickettsia* spp., *Bs Borreliella* spp. Asterisk (*) marks the significant differences in observed prevalence values or observed and expected values (*Type of area* in **a**, **b**, and **d** or infection status in **c**)

Among the tested factors, *Type of area* had an independent effect on the prevalence of Rickettsiales infections in ticks (*Type of area* × *Rickettsiales presence*/*absence*: χ^2^ = 37.8; *df* = 1; *P* < 0.001). Twice higher prevalence was detected in urban areas in comparison to natural ones (Fig. 1a).

In the second model, factor *Site* had a significant effect on Rickettsiales prevalence (*Site* × *Rickettsiales presence*/*absence*: χ^2^ = 73.3; *df* = 5; *P* < 0.001). The highest Rickettsiales prevalence was recorded in ticks from WBF—16.3%, which was also quite high in KNP—14.6% (18/105), where the lowest number of ticks was screened for pathogen DNA. The third lower prevalence was registered in other urban sites and was half lower in ticks from natural sites (Suppl. File. 3).

**Fig. 2:**
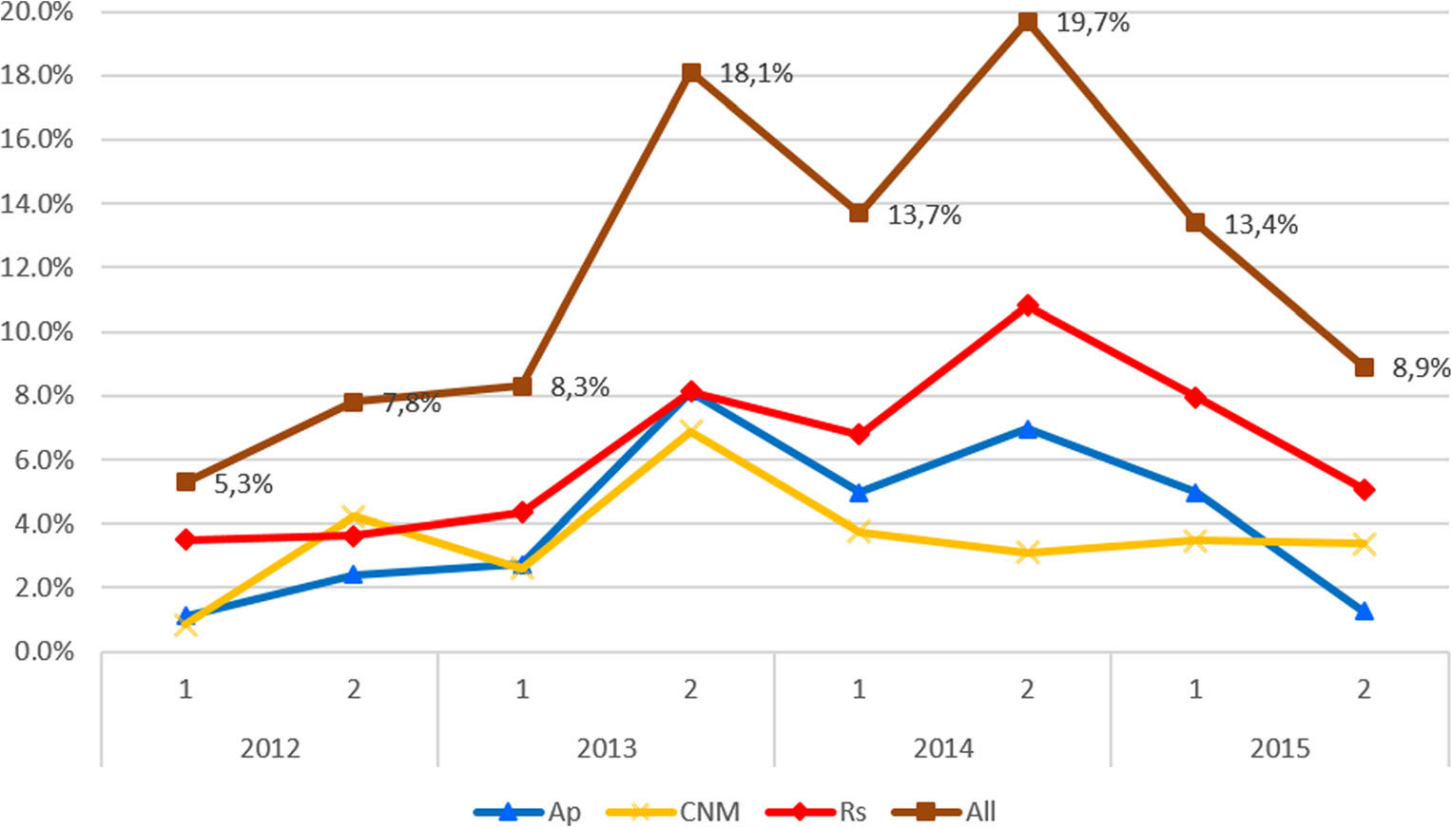
Seasonal changes of the Rickettsiales prevalence in total ticks (nymphs and adults) during 2012–2015 period. 1 spring-summer season. 2 summer-autumn season. Abbreviations: *Ap Anaplasma phagocytophilum*, *CNM* “*Candidatus* Neoehrlichia mikurensis,” *Rs Rickettsia* spp.

Additionally, the interaction of the effects of *Year* and *Season* on Rickettsiales prevalence was found to be highly significant (*Year* × *Season* × *Rickettsiales presence*/*absence*: χ^2^ = 18.0; *df* = 3; *P* < 0.001). Marked differences in the prevalence were observed depending on the year and season of study. The prevalence increased from a minimum in spring-summer in 2012 (5%) to a maximum in summer-autumn in 2014 (20%). More Rickettsiales infections were noted in ticks collected in a summer-autumn season than in spring-summer during 2012–2014, yet in 2015, the pattern was reversed (Fig. 2). The prevalence dynamics for each bacteria species followed generally the same pattern (Fig. 2). Due to these fluctuations, no independent effect of *Year* or *Season* was found.

In both subtypes of area, forests and parks, the prevalence of Rickettsiales was almost identical—14.4% and 13.5%, respectively (NS).

### *Rickettsia* spp. Infections

Overall, 5.6% (234/4189) of ticks were infected with *Rickettsia* spp.: 8.9% (181/2030) of adults and 23.8% (53/223) of the pools of nymphs were positive (MIR = 2.5%).

*Type of area* had a significant effect on the prevalence of *Rickettsia* spp. infections in ticks (*Type of area* × *Rs presence*/*absence*: χ^2^ = 6.5; *df* = 1; *P* = 0.011). More ticks were infected with these bacteria in urban areas (6.5%) than in natural ones (4.4%) (Fig. 1a).

The highest prevalence was noted in ticks from the natural forest KNP near Warsaw (13.9%) and the lowest in the natural BNP (3.2%) (*Site* × *Rs presence*/*absence*: χ^2^ = 32.9; *df* = 5; *P* < 0.001) (Table 1, Suppl. File 3). In both subtypes, forests and parks, the prevalence of *Rickettsia* spp. in ticks was very similar (5.7% vs. 5.0%; NS; Table 1).

**Table 1:** *Rickettsia* spp. prevalence in *I*. *ricinus* ticks in natural and urban sites (2012–2015)

*Rickettsia* spp.																	
*Year*			2012			2013			2014			2015			Total		
Type of area	Subtype of area	Site (particular site)	Adults	Nymphs	Total	Adults	Nymphs	Total	Adults	Nymphs	Total	Adults	Nymphs	Total	Adults	Nymphs	Total
Urban	Forest	1. WBF	0% (0/21)	4.9% (3/61)	3.7% (3/82)	12.9% (17/132)	1.8% (9/501)	4.1% (26/633)	12.5% (32/257)	4.8% (3/62)	11% (35/319)	10.8% (8/74)	6% (6/100)	8% (14/174)	11.8% (57/484)	2.9% (21/724)	6.5% (78/1208)
	Forest	2. WKF	0% (0/7)	0% (0/7)	0% (0/14)	14.3% (18/126)	1.4% (3/213)	6.2% (21/339)	12.9% (13/101)	3.6% (1/28)	10.9% (14/129)	13.8% (11/80)	5% (5/100)	8.9% (16/180)	13.4% (42/314)	2.6% (9/348)	7.7% (51/662)
	Park	3. WLP	nd	nd	nd	6.9% (11/160)	0% (0/106)	4.1% (11/266)	8.2% (8/98)	5% (1/20)	7.6% (9/118)	6% (5/83)	0% (0/26)	4.6% (5/109)	7% (24/341)	0.7% (1/152)	5.1% (25/493)
	Total		0% (0/28)	4.4% (3/68)	3.1% (3/96)	11% (46/418)	1.5% (12/820)	4.7% (58/1238)	11.6% (53/456)	4.5% (5/110)	10.2% (58/566)	10.1% (24/237)	4.9% (11/226)	7.6% (35/463)	10.8% (123/1139)	2.5% (31/1224)	6.5% (154/2363)
Natural	Total		6.6% (23/350)	1.2% (5/432)	3.6% (28/782)	5.6% (6/107)	4.6% (11/241)	4.9% (17/348)	6.3% (22/347)	2.3% (4/172)	5% (26/519)	8% (7/87)	2.2% (2/90)	5.1% (9/177)	6.5% (58/891)	2.4% (22/935)	4.4% (80/1826)
	Forest and Park	4. BNP	5.2% (13/249))	1% (3/302)	2.9% (16/551	1.5% (1/67)	1.9% (2/105)	1.7% (3/172)	4.1% (11/270)	1.9% (2/107)	3.4% (13/377)	8% (7/87)	2.2% (2/90)	5.1% (9/177)	4.8% (32/673)	1.5% (9/604)	3.2% (41/1277)
	Forest	BNW + BSW	5.8% (13/225)	1% (3/302)	3% (16/527)	2.3% (1/43)	2.1% (2/96)	2.2% (3/139)	3.8% (9/239)	1.9% (2/107)	3.2% (11/346)	8.6% (6/70)	2.2% (2/90)	5% (8/160)	5% (29/577)	1.5% (9/595)	3.2% (38/1172)
	Park	BPP	0% (0/24)	nc	0% (0/24)	0% (0/24)	0% (0/9)	0% (0/33)	6.5% (2/31)	nc	6.5% (2/31)	5.9% (1/17)	nc	5.9% (1/17)	3.1% (3/96)	0% (0/9)	2.9% (3/105)
	Forest	5. KNP	15.6% (5/32)	nc	15.6% (5/32)	12.5% (2/16)	12.5% (3/24)	12.5% (5/40)	16.2% (6/37)	7.7% (1/13)	14% (7/50)	nd	nd	nd	15.3% (13/85)	10.8% (4/37)	13.9% (17/122)
	Forest	6. MLP	7.2% (5/69)	1.5% (2/130)	3.5% (7/199)	12.5% (3/24)	5.4% (6/112)	6.6% (9/136)	12.5% (5/40)	1.9% (1/52)	6.5% (6/92)	nd	nd	nd	9.8% (13/133)	3.1% (9/294)	5.2% (22/427)
Total			6.1% (23/378)	1.6% (8/500)	3.5% (31/878)	9.9% (52/525)	2.2% (23/1061)	4.7% (75/1586)	9.3% (75/803)	3.2% (9/282)	7.7% (84/1085)	9.6% (31/324)	4.1% (13/316)	6.9% (44/640)	8.9% (181/2030)	2.5% (53/2159)	5.6% (234/4189)
Urban and Natural	Forests		5.9% (21/354)	1.6% (8/500)	3.4% (29/854)	12% (41/341)	2.4% (23/946)	5.0% (64/1287)	9.6% (65/674)	3.1% (8/262)	7.8% (73/936)	11.2% (25/224)	4.5% (13/290)	7.4% (38/514)	9.5% (152/1593)	2.6% (52/1998)	5.7% (204/3591)
	Parks		8.3% (2/24)	0% (0/0)	8.3% (2/24)	6% (11/184)	0% (0/115)	3.7% (11/299)	7.8% (10/129)	5% (1/20)	7.4% (11/149)	6% (6/100)	0% (0/26)	4.8% (6/126)	6.2% (27/437)	0.6% (1/161)	5.0% (30/598)

*Abbreviations*: *nc* not calculable (zero samples), *nd* no data (no sampling)

Additionally, we found a significant effect of *Year* on the prevalence of *Rickettsia* spp. in ticks (*Year* × *Rs presence*/*absence*: χ^2^ = 18.2; *df* = 3; *P* < 0.001). The prevalence of *Rickettsia* spp. increased from 2013 and was twice as high in 2014 and 2015 as in 2012 (Table 1). Although interactional effect of *Year* and *Season* on Rs prevalence was not significant, the prevalence fluctuated from season to season following the total Rickettsiales dynamics pattern (Fig. 2).

Among the 234 *Rickettsia* spp. positive samples, 110 *gltA* sequences were obtained. After aligning in MEGA 6.0 and a manual inspection of chromatograms, a 708-bp-long alignment was subjected to a BLAST-NCBI search. The majority of sequences (*n* = 105) were identical (100% of similarity) to *R*. *helvetica* C9P9 (GenBank accession: U59723). Our representative *R*. *helvetica* sequences (*n* = 18) derived from all study sites were deposited in GenBank (MH018961-78). Additionally, four sequences identical with the *R*. *monacensis* strain from Munich (LN794217) were recognized (our submission: MH018979-82). One of these was from the natural forest in Białowieża (BNP), while three originated from ticks from an urban forest (WBF).

The remaining single *Rickettsia gltA* sequence (our submission: KT834984) obtained from a male tick from WBF displayed only 85% similarity to our *R*. *monacensis* sequences and 86% sequence homology to our *R*. *helvetica* sequences. It was identical with *gltA* sequences of recently described “*Candidatus* R. mendelii” (N849396, KJ882309, and KJ882311) from *I*. *ricinus* ticks detached from songbirds and questing ticks from the Czech Republic.

### Anaplasmataceae Infections

Overall, 5.8% (242/4189) of ticks were infected with either Ap or CNM (any Anaplasmataceae). The prevalence of Anaplasmataceae was 8.6% in adults (175/2030) and 30.0% (67/223) in the pools of nymphs (MIR = 3.1%).

Overall, at least 3.5% (146/4189) of ticks were infected with Ap. The prevalence of Ap infection in adult ticks was 6.0% (121/2030). Only 11.2% (25/223) of the pools of nymphs were infected with Ap (MIR = 2.2%). *Type of area* had a significant effect on the prevalence of Ap infection in ticks (*Type of area* × *Ap presence*/*absence*: χ^2^ = 59.0; *df* = 1; *P* < 0.001). More ticks were infected with Ap in urban areas than in natural areas (5.3% vs. 1.1%, respectively) (Fig. 1a). Accordingly, the effect of *Site* on Ap prevalence was also significant (*Site* × *Ap presence*/*absence*: χ^2^ = 133.9; *df* = 5; *P* < 0.001). The highest prevalence of Ap was recorded in an urban forest WBF and an urban park WLP (4–8%; Table 2, Suppl. File 3). The lowest prevalence was found in an urban forest WKF and a natural forest MLP (0.6–0.7%; Table 2, Suppl. File 3).

**Table 2:** *Anaplasma phagocytophilum* in *I*. *ricinus* ticks in natural and urban sites (2012–2015)

*Anaplasma phagocytophilum*																	
*Year*			2012			2013			2014			2015			Total		
Type of area	Subtype of area	Site (particular site)	Adults	Nymphs	Total	Adults	Nymphs	Total	Adults	Nymphs	Total	Adults	Nymphs	Total	Adults	Nymphs	Total
Urban	Forest	1. WBF	9.5% (2/21)	3.3% (2/61)	4.9% (4/82)	22% (29/132)	1.8% (9/501)	6% (38/633)	16.3% (42/257)	6.5% (4/62)	14.4% (46/319)	13.5% (10/74)	4% (4/100)	8% (14/174)	17.1% (83/484)	2.6% (19/724)	8.4% (102/1208)
	Forest	2. WKF	0% (0/7)	0% (0/7)	0% (0/14)	0% (0/126)	0% (0/213)	0% (0/339)	2% (2/101)	0% (0/28)	1.6% (2/129)	1.3% (1/80)	1% (1/100)	1.1% (2/180)	1% (3/314)	0.3% (1/348)	0.6% (4/662)
	Park	3. WLP	nd	nd	nd	6.3% (10/160)	0% (0/106)	3.8% (10/266)	5.1% (5/98)	0% (0/20)	4.2% (5/118)	4.8% (4/83)	3.8% (1/26)	4.6% (5/109)	5.6% (19/341)	0.7% (1/152)	4.1% (20/493)
	Total		7.1% (2/28)	2.9% (2/68)	4.2% (4/96)	9.3% (39/418)	1.1% (9/820)	3.9% (48/1238)	10.7% (49/456)	3.6% (4/110)	9.4% (53/566)	6.3% (15/237)	2.7% (6/226)	4.5% (21/463)	9.2% (105/1139)	1.7% (21/1224)	5.3% (126/2363)
Natural	Total		2% (7/350)	0.2% (1/432)	1% (8/782)	0.9% (1/107)	1.2% (3/241)	1.1% (4/348)	1.7% (6/347)	0% (0/172)	1.2% (6/519)	2.3% (2/87)	0% (0/90)	1.1% (2/177)	1.8% (16/891)	0.4% (4/935)	1.1% (20/1826)
	Forest and Park	4. BNP	2.8% (7/249)	0.3% (1/302)	1.5% (8/551)	0% (0/67)	0% (0/105)	0% (0/172)	1.9% (5/270)	0% (0/107)	1.3% (5/377)	2.3% (2/87)	0% (0/90)	1.1% (2/177)	2.1% (14/673)	0.2% (1/604)	1.2% (15/1277)
	Forest	BNW+ BSW	3.1% (7/225)	0.3% (1/302)	1.5% (8/527)	0% (0/43)	0% (0/96)	0% (0/139)	2.1% (5/239)	0% (0/107)	1.4% (5/346)	2.9% (2/70)	0% (0/90)	1.3% (2/160)	2.4% (14/577)	0.2% (1/595)	1.3% (15/1172)
	Park	BPP	0% (0/24)	nc	0% (0/24)	0% (0/24)	0% (0/9)	0% (0/33)	0% (0/31)	nc	0% (0/31)	0% (0/17)	nc	0% (0/17)	0% (0/96)	0% (0/9)	0% (0/105)
	Forest	5. KNP	0% (0/32)	nc	0% (0/32)	0% (0/16)	4.2% (1/24)	2.5% (1/40)	2.7% (1/37)	0% (0/13)	2% (1/50)	nd	nd	nd	1.2% (1/85)	2.7% (1/37)	1.6% (2/122)
	Forest	6. MLP	0% (0/69)	0% (0/130)	0% (0/199)	4.2% (1/24)	1.8% (2/112)	2.2% (3/136)	0% (0/40)	0% (0/52)	0% (0/92)	nd	nd	nd	0.8% (1/133)	0.7% (2/294)	0.7% (3/427)
Total			2.4% (9/378)	0.6% (3/500)	1.4% (12/878)	7.6% (40/525)	1.1% (12/1061)	3.3% (52/1586)	6.8% (55/803)	1.4% (4/282)	5.4% (59/1085)	5.2% (17/324)	1.9% (6/316)	3.6% (23/640)	6% (121/2030)	1.2% (25/2159)	3.5% (146/4189)
Urban and Natural	Forests		2.5% (9/354)	0.6% (3/500)	1.4% (12/854)	8.8% (30/341)	1.3% (12/946)	3.3% (42/1287)	7.4% (50/674)	1.5% (4/262)	5.8% (54/936)	5.8% (13/224)	1.7% (5/290)	3.5% (18/514)	6.4% (102/1593)	1.2% (24/1998)	3.5% (126/3591)
	Parks		0% (0/24)	0% (0/0)	0% (0/24)	5.4% (10/184)	0% (0/115)	3.3% (10/299)	3.9% (5/129)	0% (0/20)	3.4% (5/149)	4% (4/100)	3.8% (1/26)	4% (5/126)	4.3% (19/437)	0.6% (1/161)	3.3% (20/598)

*Abbreviations*: *nc* not calculable (zero samples), *nd* no data (no sampling)

No independent effect of *Subtype of area* on Ap prevalence was found (Table 2); however, the interaction of effects of *Season* and *Subtype of area* affected Ap prevalence in ticks (*Season* × *Subtype of area* × *Ap presence*/*absence*: χ^2^ = 4.0; *df* = 1; *P* = 0.046). The prevalence of Ap in ticks was similarly low in both parks and forests in the spring-summer season, but over twice as high in parks in comparison to forests in summer-autumn (Suppl. File 4).

Additionally, interaction of *Year* and *Season* effects was found to be significant for Ap prevalence (*Year* × *Season* × *Ap presence*/*absence*: χ^2^ = 21.7; *df* = 3; *P* < 0.001). Generally, in the summer-autumn period, more ticks were infected with Ap than in spring-early summer, except in 2015, when the pattern was reversed (Fig. 2).

The 16S rDNA fragment of eight Ap-positive samples from WBF was sequenced to confirm the specificity of Ap PCR detection by BLAST analysis. All sequences in the 862 bp alignment revealed highest homology to *A*. *phagocytophilum*, with the identity of 99.8% to 100%. One sequence (MH122884) was identical with the Ap sequences derived from *I*. *ricinus* from Belarus (HQ629914), from a tick, and *Alces alces* spleen from Sweden (AJ242783, KC800983). The remaining sequences (MH122885-91) were identical with Ap sequences obtained from a dog in Germany (JX173651), *I*. *ricinus* from Belarus and Russia (HQ629915, HQ629911), and even a raccoon dog from South Korea (KY458571).

In total, only 2.9% (122/4189) of ticks were infected with CNM. The prevalence of CNM was 3.5% (71/2030) in adults, while in the pools of nymphs, it was 22.9% (MIR = 2.4% (51/2159) (Table 3). In contrast to Ap, *Type of area* had no significant effect on CNM prevalence in ticks, although numerically overall CNM prevalence was again lower in natural areas in comparison to urban areas (Table 3).

**Table 3:** “*Candidatus* Neoehrlichia mikurensis” prevalence in *I*. *ricinus* ticks in natural and urban sites (2012–2015)

“Candidatus Neoehrlichia mikurensis”																	
Year			2012			2013			2014			2015			Total		
Type of area	Subtype of area	Site (particular site)	Adults	Nymphs	Total	Adults	Nymphs	Total	Adults	Nymphs	Total	Adults	Nymphs	Total	Adults	Nymphs	Total
Urban	Forest	1. WBF	0% (0/21)	8.2% (5/61)	6.1% (5/82)	6.1% (8/132)	3.6% (18/501)	4.1% (26/633)	7% (18/257)	4.8% (3/62)	6.6% (21/319)	6.8% (5/74)	3% (3/100)	4.6% (8/174)	6.4% (31/484)	4% (29/724)	5% (60/1208)
	Forest	2. WKF	0% (0/7)	0% (0/7)	0% (0/14)	3.2% (4/126)	0.9% (2/213)	1.8% (6/339)	3% (3/101)	3.6% (1/28)	3.1% (4/129)	2.5% (2/80)	1% (1/100)	1.7% (3/180)	2.9% (9/314)	1.1% (4/348)	2% (13/662)
	Park	3. WLP	nd	nd	nd	3.1% (5/160)	0% (0/106)	1.9% (5/266)	0% (0/98)	0% (0/20)	0% (0/118)	7.2% (6/83)	0% (0/26)	5.5% (6/109)	3.2% (11/341)	0% (0/152)	2.2% (11/493)
	Total		0% (0/28)	7.4% (5/68)	5.2% (5/96)	4.1% (17/418)	2.4% (20/820)	3% (37/1238)	4.6% (21/456)	3.6% (4/110)	4.4% (25/566)	5.5% (13/237)	1.8% (4/226)	3.7% (17/463)	4.5% (51/1139)	2.7% (33/1224)	3.6% (84/2363)
Rural	Total		1.7% (6/350)	0.5% (2/432)	1% (8/782)	0.9% (1/107)	4.1% (10/241)	3.2% (11/348)	2.6% (9/347)	2.9% (5/172)	2.7% (14/519)	4.6% (4/87)	1.1% (1/90)	2.8% (5/177)	2.2% (20/891)	1.9% (18/935)	2.1% (38/1826)
	Forest and Park	4. BNP	0.8% (2/249)	0.3% (1/302)	0.5% (3/551)	0% (0/67)	3.8% (4/105)	2.3% (4/172)	2.6% (7/270)	2.8% (3/107)	2.7% (10/377)	4.6% (4/87)	1.1% (1/90)	2.8% (5/177)	1.9% (13/673)	1.5% (9/604)	1.7% (22/1277)
	Forest	BNW + BSW	0.9% (2/225)	0.3% (1/302)	0.6% (3/527)	0% (0/43)	4.2% (4/96)	2.9% (4/139)	2.9% (7/239)	2.8% (3/107)	2.9% (10/346)	4.3% (3/70)	1.1% (1/90)	2.5% (4/160)	2.1% (12/577)	1.5% (9/595)	1.8% (21/1172)
	Park	BPP	0% (0/24)	nc	0% (0/24)	0% (0/24)	0% (0/9)	0% (0/33)	0% (0/31)	nc	0% (0/31)	5.9% (1/17)	nc	5.9% (1/17)	1% (1/96)	0% (0/9)	1% (1/105)
	Forest	5. KNP	0% (0/32)	nc	0% (0/32)	0% (0/16)	8.3% (2/24)	5% (2/40)	0% (0/37)	0% (0/13)	0% (0/50)	nd	nd	nd	0% (0/85)	5.4% (2/37)	1.6% (2/122)
	Forest	6. MLP	5.8% (4/69)	0.8% (1/130)	2.5% (5/199)	4.2% (1/24)	3.6% (4/112)	3.7% (5/136)	5% (2/40)	3.8% (2/52)	4.3% (4/92)	nd	nd	nd	5.3% (7/133)	2.4% (7/294)	3.3% (14/427)
Total			1.6% (6/378)	1.4% (7/500)	1.5% (13/878)	3.4% (18/525)	2.8% (30/1061)	3% (48/1586)	3.7% (30/803)	3.2% (9/282)	3.6% (39/1085)	5.2% (17/324)	1.6% (5/316)	3.4% (22/640)	3.5% (71/2030)	2.4% (51/2159)	2.9% (122/4189)
Urban and Natural	Forests		1.4% (5/354)	1.4% (7/500)	1.4% (12/854)	3.8% (13/341)	3.2% (30/946)	3.3% (43/1287)	4.5% (30/674)	3.4% (9/262)	4.2% (39/936)	4.5% (10/224)	1.7% (5/290)	2.9% (15/514)	3.6% (58/1593)	2.6% (51/1998)	3% (109/3591)
	Parks		4.2% (1/24)	0% (0/0)	4.2% (1/24)	2.7% (5/184)	0% (0/115)	1.7% (5/299)	0% (0/129)	0% (0/20)	0% (0/149)	7% (7/100)	0% (0/26)	5.6% (7/126)	3% (13/437)	0% (0/161)	2.2% (13/598)

*Abbreviations*: *nc* not calculable (zero samples), *nd* no data (no sampling)

There was a significant effect of *Site* on CNM prevalence in ticks (*Site* × *CNM presence*/*absence*: χ^2^ = 23.9; *df* = 5; *P* < 0.001). The highest prevalence of CNM infections was found in the urban forest WBF and was also quite high in the natural forest MLP, in comparison to other urban or natural sites (Suppl. File 3).

Also, *Subtype of area* affected CNM prevalence in different ways, depending on the year of the study (*Year* × *Subtype of area* × *CNM presence*/*absence*: χ^2^ = 15.6; *df* = 3; *P* = 0.001). In 2012 and 2015, more ticks were infected in parks than in forests; the opposite pattern was recorded in 2013 and 2014. The 4-year total CNM prevalence was very similar in both subtypes of area (3% vs. 2.2%) (Table 3).

As for Ap prevalence, the interactional effect of *Year* and *Season* on the prevalence of CNM was significant (*Year* × *Season* × *CNM presence*/*absence*: χ^2^ = 10.8; *df* = 3; *P* = 0.013). Generally, ticks were more infected with CNM in the summer-autumn season in 2012 and 2013; however, the prevalence was comparable in both seasons in 2014 and 2015 (Fig. 2).

The 16S rDNA fragment of 10 randomly selected CNM-positive samples was sequenced and compared with GenBank-deposited sequences through BLAST analysis. All sequences in the 422 bp 16S rDNA fragment alignment were either identical (our submissions: MH122892, MH122894-901) or almost identical (99.8%) (MH122893) with the CNM Nagano21 strain derived from *Apodemus speciosus* from Japan (AB196305) and with numerous European and Asian isolates (e.g., MF351959 from South Korea, FJ966360 from Russia, or AF104680 from The Netherlands).

### Co-infections

A total of 2.7% (60/2253) of samples were positive for at least two of the investigated bacteria. Due to the uncertainty of the co-infection status in the pools of nymphs, these samples were excluded from the co-infection analyses. Among the adult ticks, 1.6% were co-infected (33/2030), in comparison with the expected co-infection frequency of 1.1%. Co-infections were most common in the Warsaw area, at WBF and WLP (5.0 and 1.2%, respectively). In WKF and MLP, the prevalence of co-infections was similar (0.6 and 0.8%, respectively). In BNP, only 0.3% of adults were co-infected (*Site* × *any Rickettsiales co*-*infection presence*/*absence*: χ2 = 37.8, *df* = 1, *P* < 0.001). No co-infections were recorded in KNP.

Among the tested factors, *Type of area* had an independent effect on co-infection prevalence (*Type of area* × *any Rickettsiales co*-*infection presence*/*absence*: χ^2^ = 12.6, *df* = 1, *P* < 0.001). The observed prevalence of co-infections between any Rickettsiales was significantly higher in urban areas, in comparison to natural areas (2.6% vs. 0.3%, respectively). The prevalence value was higher than expected in urban areas, while no difference between expected and observed frequency was noted in natural areas (Fig. 1b).

*Type of area* also had a significant effect on the prevalence of particular co-infections: for “Ap and CNM” (*Type of area* × “Ap and CNM” *presence*/*absence*: χ^2^ = 10.1, *df* = 1, *P* < 0.001) and “Ap and Rs” (*Type of area* × “Ap and Rs” *presence*/*absence*: χ^2^ = 9.9, *df* = 1, *P* = 0.002). In urban areas, these co-infections were more common than in natural areas (Fig. 1b). None of the investigated factors affected the occurrence of “CNM and Rs” co-infections.

Additionally, the interaction of the effect of *Year* and *Season* was found to influence the occurrence of Rickettsiales co-infections in adult ticks: very low percentages of co-infections were found every year, particularly little or no co-infections were recorded in summer-autumn season. The exception being in 2013 when the highest prevalence of co-infections (8.4%) was noted in the summer-autumn season (*Year* × *Season* × *any co*-*infection presence*/*absence*: χ^2^ = 18.0, *df* = 1, *P* < 0.001) (Suppl. File 5). When analyzing two-species co-infections in particular, this effect interaction was only significant for the “Ap and Rs” combination (*Year* × *Season* × “Ap and Rs” *presence*/*absence*: χ^2^ = 9.4, *df* = 1, *P* < 0.025). Although no such co-infections were detected in 2012 and 2015, in 2013, the prevalence of “Ap and Rs” co-infection was seven times higher in the summer-autumn season than in the spring-summer (Suppl. File 5).

Co-infections between Anaplasmataceae family members were the most common in *I*. *ricinus* ticks: the observed prevalence of co-infection of “Ap and CNM” was 0.8% and it was four times higher than the expected value (0.2%; Ap presence/absence × CNM presence/absence: χ^2^ = 25.9; *df* = 1; *P* < 0.001). Also, more “Ap and CNM” co-infections were observed in urban areas than in natural areas (1.4% vs. 0.1%), over three times the expected value (0.4%) (*Type of area* × “Ap and CNM” *presence*/*absence*: χ^2^ = 10.1; *df* = 1; *P* < 0.001) (Fig. 1b).

The positive association between the occurrence of Ap and CNM infections was finally confirmed by comparison of the prevalence of one species between two groups of ticks: infected and uninfected with the second species (Fig. 1c). Interestingly, in both types of areas, the prevalence of certain pathogen (Ap or CNM) was four times higher in group infected with the second one (either Ap or CNM), e.g., the prevalence of Ap reached 31% in a group of CNM-positive ticks in urban areas, while only 8% in CNM-negative ticks (*P* < 0.001).

The observed prevalence of rare co-infections (“Ap and Rs,” “CNM and Rs”) did not differ from expected values; there was no significant effect of Rs infection on Ap or CNM prevalence. The overall prevalence of “Ap and Rs” co-infection was 0.6% in adult ticks (expected 0.5%, NS), while “CNM and Rs” was only 0.2% (expected 0.3%, NS).

### Rickettsiales and Spirochaetes Co-infections

*Season* and *Type of area* had significant independent effect on the prevalence of “Rickettsiales and spirochaetes” co-infections (*Type of area* × *any* “*Rickettsiales and spirochaetes*” *presence*/*absence*: χ^2^ = 12.0, *df* = 1, *P* = 0.001; *Season* × *any* “*Rickettsiales and spirochaetes*” *presence*/*absence*: χ^2^ = 5.6, *df* = 1, *P* = 0.018). More observed “Rickettsiales and spirochaetes” co-infections were recorded in urban areas than were recorded in natural areas (5.0% vs. 1.5%) (Fig. 1d). Also, over twice as high prevalence of co-infection of this kind was detected in the second season, summer-autumn, as in the first one (6.5% vs. 3.1%).

Among the analyzed factors, only *Type of area* affected the observed prevalence of the “Rs and spirochaetes” co-infection (*Type of area* × “*Rs and spirochaetes*” *presence*/*absence*: χ^2^ = 8.2, *df* = 1, *P* = 0.004). Such co-infections were four times more frequent in urban than in natural areas (2.7% vs. 0.7%, respectively).

Only *Season* affected the prevalence of “Ap and spirochaetes” co-infection in ticks (*Season* × “*Ap and spirochaetes*” *presence*/*absence*: χ^2^ = 4.8, *df* = 1, *P* = 0.028). Such co-infections were almost three times more frequent in summer-autumn than in spring-summer (2.5% vs. 0.9%, respectively).

No factors influenced the prevalence of “CNM and spirochaetes” co-infections in adult ticks. No particular “Rickettsiales and spirochaetes” co-infection occurred significantly more frequently than was expected from a random co-occurrence in ticks (Fig. 1d).

## Discussion

In this study, we have found that Rickettsiales infections and co-infections occur significantly more often in *I*. *ricinus* ticks from urban areas in comparison to natural areas. All pathogens investigated (Rs, Ap, and CNM) have shown similar or identical trends. More importantly, the same pattern was observed for co-infections of almost every investigated pathogen: significantly higher prevalence of co-infections in adult *I*. *ricinus* ticks was observed in urban areas (except for “Rs and CNM”). Additionally, also *Year* and *Season* seemed to influence the Rickettsiales prevalence in ticks, showing the long-term dynamics of the Rickettsiales prevalence. Finally, using the data obtained in our previous study [24], we analyzed the prevalence of co-infection of selected Rickettsiales with *Borreliella* spp. spirochaetes. The fraction of co-infection of Rickettsiales and spirochaetes in adult ticks was over twice as high in urban as in natural areas.

In this complex long-term study, we determined the prevalence of three Rickettsiales species in questing *I*. *ricinus* ticks from different habitats. Although the prevalence of individual species was rather low (highest for total *Rickettsia* spp., lowest for CNM), below 6%, the total prevalence of Rickettsiales infection achieved 10% (MIR) in the total number of tested ticks but up to 17% in individually processed adults. Thus, these Rickettsiales infections seem to constitute an important part of the tick pathogen community in Poland.

For the estimation of the total prevalence in ticks (adults and nymphs), MIR was calculated; thus, the overall prevalence may be underestimated in the case of where more than one nymph in the pool was infected. Thus, a more useful generalization can be derived from the analysis of the prevalence in adult ticks. But also in adult ticks, there is a similar clear pattern: the most common were infections with Rs, then Ap, and the rarest with CNM (Tables 1, 2, and 3). Interestingly, this prevalence hierarchy was maintained in the majority of seasons during the 4-year-period (Fig. 2). The prevalence varied also between sites, but the highest Rickettsiales prevalence was observed in the urban forest WBF, due to a relatively high prevalence of Ap, CNM, and Rs (Suppl. File 3). The second highest prevalence of Rickettsiales was observed in the natural forest KNP, localized in the Warsaw vicinity, due to the highest prevalence of Rs. Thus, overall, it seems that Rs prevalence is higher not only in urban sites but generally in Mazovia (central Poland) in comparison to the less-changed NE regions of Poland (Białowieża, Mazury lake district).

Relatively low tick abundance was recorded in both KNP and MLP (Suppl. File 2), in comparison with the abundance in Warsaw city forests/park and Białowieża forests [24, 29]. In MLP, the abundance of ticks was very similar to natural BPP or urban WKF and WLP. It was also half as low in KNP in comparison to these sites, sustaining the findings of our previous study, that KNP and WKF are forested areas of actually low tick-abundance status [29].

Despite generally lower tick abundance in the studied urban areas in comparison to natural areas in Białowieża [24] or no differences in abundance between urban areas and natural areas of NE and central Poland in this study, the prevalence of Rickettsiales was twice as high in urban as natural areas. This effect is particularly well observed when comparing the prevalence of infection in individually processed adult ticks. Prevalence values are twice as high for every bacteria species: for Ap 9.2% in urban vs. 1.8% in natural, for Rs 11% in urban vs. 6.5% in natural, for CNM 4.5% in urban vs. 2.2% in natural. Although prevalence tended to be higher in forests than in parks (particularly in adult ticks; Tables 1, 2, and 3), this resulted mainly from very low prevalence values in ticks from BPP (a natural area), but in a Warsaw park (WLP), prevalence was relatively high (Suppl. File 3). Thus, among Warsaw habitats, the highest risk of contracting rickettsiae seems to exist either in a city forest (WBF) or city park (WLP).

There was no clear seasonal pattern for the Rickettsiales prevalence in ticks, mainly due to significant differences between years. However, in 2 years out of 4, the prevalence was higher in the second season of tick activity (summer-autumn) despite the lower abundance of ticks in that season ([24]; Fig. 2). Thus, it seems that higher prevalence in the summer-autumn season is due to cumulating infections from infected hosts. In our long-term study on vector-borne parasites in bank voles in the same area [36, 37], the highest prevalence of infections in voles occurred in summer or autumn, due to higher vector activity in a warmer time of the year; thus, the same mechanism may be the cause of increased prevalence in ticks following feeding on their infected hosts.

In this study, we determined and analyzed expected and observed co-infections, including co-infections among Rickettsiales species and co-infections between Rickettsiales and *Borreliella* spp. Not surprisingly, co-infections involving rickettsiae and spirochaetes were more common (5% of adult *I*. *ricinus*) than co-infections among Rickettsiales (overall 2.6%) due to a higher prevalence of *Borreliella* spp. in ticks [24]. Interestingly, co-infections were not only more common in urban areas (due to a generally higher percentage of rickettsiae-infected ticks in urban areas) but also tended to be more common than expected prevalence (Fig. 1b, d). Among Rickettsiales, there was positive association between Ap and CNM occurrence, resulting in high prevalence of CNM (rare species: 3.5% in adult ticks) in Ap-infected ticks (15%) (Fig. 1c). These findings underlined a relatively high risk of contracting mixed infections by a single tick bite in urbanized areas.

### Prevalence of Rickettsiales Infections and Co-infections

#### *Rickettsia* spp.

In comparison to other European studies, we have found a relatively low prevalence of infection with Rs in ticks: 5.6% (including MIR in nymphs). In Europe, the prevalence of Rs in ticks varies greatly, depending on study location, from only 0.5% to even 66% [38, 39]; reviewed in [1]. In a recent study on ticks from the Białowieża region conducted in 2013, authors reported the prevalence of *Rickettsia* spp. varying locally from 3.3% to as high as 27.8%. Overall, 8.6% of tested samples (based also on MIR) were *Rickettsia*-positive [40], twice as high as in our study (4.4%). The same authors also reported quite high prevalence of Rs infection in KNP (in 2012 and 2013) in comparison to our data (27.5% vs. 13.9%, respectively), higher than previously reported in KNP [41].

In our study, the prevalence of Rs in questing ticks was significantly higher in urban areas, although still relatively low: 6.5%. Similar prevalence was reported in recreational sites in urban areas of Bavaria, Germany (6.4–7.7%) [42], Bratislava (7.8%) [43], and Paris (5.8%) [44]. Very similar values were obtained in our previous study in 2011, when the prevalence in Warsaw (urban areas) was 7.7% in comparison to 2.9% in natural areas [29].

The dominant *Rickettsia* species was *R*. *helvetica*, which is in concordance with numerous European studies confirming the common presence of this tick-parasitic bacteria in *I*. *ricinus* [1]. Much rarer was *R*. *monacensis* infection. The presence of both *Rickettsia* species was reported in our previous study [29]. The third *Rickettsia* species detected in this study was novel “*Ca*. R. mendelii.” It was detected for the first time in Poland in the first year of this study (2013) in a tick from WBF and deposited as *Rickettsia* sp. IrLB1 in the GenBank database (submitted in 2016: KT834984), before the species was described by Hajduskova et al. in 2016 [45]. Since that time, “*Ca*. R. mendelii” was also detected in *I*. *ricinus* from BNP [40] with the use of a second molecular marker (*rss*), indicating that this bacterium is present in Poland in both natural and urban areas. A further study on the potential pathogenicity and circulation of this novel rickettsia is required.

#### *Anaplasma phagocytophilum*

The prevalence of Ap varies locally from 0.4 to 20% or more in different European countries [38, 39, 46]; reviewed in [1]. In Poland, the prevalence of Ap in questing *I*. *ricinus* ticks varies from 4.9 to 23.7%, locally up to 38.5% ([29, 47–49]). In our study, the maximum local prevalence reached 16.3% in WBF in 2014, but in total, only 5.3% of ticks (including MIR) were infected. The local differences may be explained by the availability of a competent reservoir of pathogens (increasing prevalence) and non-competent as well, especially in less transformed areas with greater biodiversity, which contribute to a dilution effect (decreasing prevalence) [50]. The limitation of this study is the lack of identification of particular Ap strains. This pathogen species is considered to circulate as an assemblage of strains that differ not only by the geographic origin, but also by host specificity and thus are believed to have different pathogenicity [51]. In our previous study, the majority of strain variants detected in ticks in both urban (Warsaw) and natural areas (Białowieża) were zoonotic [29].

#### “*Candidatus* Neoehrlichia mikurensis”

The prevalence of CNM in *I*. *ricinus* ticks in our study sites (2–3%) was similar to the prevalence noted in Germany (2.2%), France (1.7%) and the Czech Republic (2.2%), and in other European countries, in which prevalence varied from 0.4 up to 26.6%, however in majority of cases, was not higher than 10% (reviewed in [1, 11, 39]). CNM occurred more often in urban than in natural areas; however, the differences were insignificant. Obtained CNM 16S rDNA sequences displayed high homology with other isolates from Europe and Asia confirming low heterogeneity of this marker [52–55].

### Rickettsial Co-infections

According to our data, there is an increased prevalence of Rickettsiales co-infections in urban than in natural areas, indicating an increased risk of infection in the case of a tick bite in city forest or recreational area. We found positive association for co-occurrence of Ap and CNM, which is in concordance with findings already published [56]. Both pathogens share not only reservoir mammal hosts, but could actually promote each other’s transmission to a vector, as in the case of Ap and Lyme spirochaetes [20]. More data is needed to confirm this phenomenon. It indicates the increased probability of co-infection with both pathogens in the case of a tick bite.

Alternatively, in a study in the Netherlands, negative association between the occurrence of *R*. *helvetica* and CNM was found [57]. Although our data did not support any effect of *Rickettsia* spp. infection on CNM infections (or any other factors), the observed prevalence of “Rs and CNM” co-infection was lower than expected. Although there were too few co-infections detected in this study (*n* = 4) to come to a conclusion, future studies may confirm whether there is indeed a negative association.

#### Prevalence of Rickettsiales and *Borreliella* spp. Spirochaetes Co-infections

Despite no significant differences between expected and observed of prevalence values in adult ticks, the Rickettsiales and *Borreliella* spp. co-infection seemed to occur slightly more frequently than is expected from random co-occurrence in urban areas and less often than expected in natural areas (Fig. 1d). In a similar study conducted on *I*. *ricinus* ticks from cities of Hanover and Hamburg, Germany, significant positive association was found between the occurrence of Rs and spirochaetes, but only in nymphs. Similar to our findings, this type of co-infection was observed only a bit more often than expected in adult ticks (NS) [58]. Because of the differences in the ecological relationship with ticks (being a reservoir or vector for these two groups of bacteria, respectively), it remains to be investigated what is the eventual mechanism of such interaction in a tick-organism and for transmission. Co-occurrence of both groups of bacteria indicates a common host [57]. This is in concordance with the hypothesis of a lower dilution effect on Rickettsiales in cities, as a result of a lower number of tick-host species, including incompetent ones. It remains to be verified with previous findings of the significant negative association between Lyme spirochaetes and *R*. *helvetica* infections [57].

In our study, both groups, adults infected and uninfected with Rickettsiales, were almost equally infected with spirochaetes. This finding cannot be directly compared with the previously reported positive association in nymphs [58] because we could not analyze the prevalence of co-infections in nymphs, due to the pooling of the specimens for screening. Because of this limitation, nymphs should be analyzed individually for co-infections in future studies, especially because this stage most commonly infests humans. The prevalence of co-infection with “Rs and *Borreliella* spp.” observed in our study (1.8%) was much lower than in Germany (12% observed vs. 9% expected) [58], but similar to the findings from Romania (1.3%) [59].

As there were no differences in the prevalence of *Borreliella* spp. in ticks from urban and natural areas [24], but there were such differences in the prevalence of Rickettsiales, it is evident that differences in the prevalence of co-infection with spirochaetes and rickettsiae result mainly from rickettsiae prevalence in ticks. And this prevalence is affected by variety of factors possibly associated with human impact. The question to be answered is what particular factors cause the *Type of area* effect.

The urbanization effect on the circulation of tick-borne pathogens was graphically shown and briefed by Rizzoli et al. [1]. The reasons of higher Rickettsiales (Rs, Ap, and CNM in our paper) prevalence in ticks observed in urban areas affected by large human population and high level of environment transformation might be various. This outcome could be connected with environmental factors like air pollution or higher level of average temperatures in cities, which may not affect tick abundance, but possibly their fitness and competence as carriers of pathogens. Also, in urban areas, there is evident fragmentation (due to infrastructure and growing suburbs) of forested areas and in many cases the isolation of tick habitats within urban, highly transformed matrix. Since the space and often access to potential tick habitats are limited for certain hosts (e.g., big ruminants), this may promote the Rickettsiales “amplification” by circulation in a limited number of tick-host species on the area of generally higher host density. On the other hand, cities attract synanthropic species of hosts: medium-sized predators or omnivores, as well as ground-foraging birds; especially in parks and small urban forests. Thus, the “structure of tick-host population” might be different in cities in many aspects in comparison to natural areas, affecting the Rickettsiales prevalence in ticks.

Our findings suggest that in urban areas, there is not only a higher risk of encountering the ticks infected with Rickettsiales bacteria, but also co-infected with Lyme spirochaetes. Conversely, despite almost identical distribution of *Borreliella* spp. in ticks in urban and natural areas, in urban areas, there is a higher risk that a tick infected with Lyme spirochaetes is co-infected with another pathogen. This may also enhance transmission of tick-borne pathogens to reservoir hosts. According to our previous study, the dominant strain of Ap in ticks in both urban (Warsaw) and natural areas (Białowieża) is likely zoonotic [29]. These facts would be important since co-infection of Ap and *Borreliella* spp. may facilitate the transmission of each pathogen from an infected host to a vector [20], mediating the possibility of an increase in prevalence in ticks in urban foci. A higher rate of such co-infection in ticks in urban areas seems to constitute a great challenge for public health; however, this phenomenon shall be further investigated.

## Conclusion

An increased level of human impact in urban areas positively affects the transmission and maintenance of Rickettsiales in the population of ticks: more ticks were infected with *A*. *phagocytophilum* and *Rickettsia* spp. in urban areas than in natural ones. High prevalence of “*Ca*. N. mikurensis” was found in ticks infected with *A*. *phagocytophilum* in both types of areas. Moreover, there is a higher risk of encountering an *I*. *ricinus* tick co-infected with other TBPs, also with Lyme spirochaetes, in the Warsaw agglomeration in comparison to natural areas. This finding raises the question whether cities might be in fact the hot spots for TBDs.

## Electronic Supplementary Material

Suppl. File 1 Classification of the study sites. (DOCX 37 kb)

Suppl. File 2 Tick abundance (mean no. of ticks per 100 m^2^ ± SE) in Kampinoski National Park (KNP) and Mazurian Landscape Park (MLP) (DOCX 14 kb)

Suppl. File 3 Prevalence of Rickettsiales in total ticks in natural and urban sites (2012–2015 average). (DOCX 15 kb)

Suppl. File 4 *Anaplasma phagocytophilum* infection prevalence in total *I*. *ricinus* ticks in two Subtypes of areas in two seasons (2012–2015 average) (DOCX 22 kb)

Suppl. File 5 Rickettsiales co-infection prevalence in adult *I*. *ricinu*s ticks urban and natural areas (DOCX 14 kb)
