# Supplementary material for: Rickettsiales Occurrence and Co-occurrence in Ixodes ricinus Ticks in Natural and Urban Areas
Source: Microb Ecol. 2018 Oct 16;77(4):890–904. doi: 10.1007/s00248-018-1269-y (PMC6478632; doi:10.1007/s00248-018-1269-y)
Supplement: Supplementary file 1 — Classification of the study sites. (DOCX 37 kb) [file 248_2018_1269_MOESM1_ESM.docx]

**Supplementary File 1** Supplementary Figure 1. Classification of the study sites.

*Subtype of area*

*Type of area*

*Site*

The scheme presents the classification of the study sites in terms of: level of human impact (*Type of area*), main localities (*Site*) in the study and particular sites in Białowieża, which represents two class of environments (*Subtype of area*): forest (full frame) and parks (dash frame). Abbreviations: *BNP* Białowieża National Park, *KNP* Kampinoski National Park, *MLP* Mazurski Landscape Park, *WBF* Warsaw – Bielański Forest, *WKF* Warsaw – Kabacki Forest, *WLP* – Warsaw – Łazienki Królewskie Park, *BNW* Białowieża – North-West, *BSW* Białowieża – South-West
