# Supplementary material for: Rickettsiales Occurrence and Co-occurrence in Ixodes ricinus Ticks in Natural and Urban Areas
Source: Microb Ecol. 2018 Oct 16;77(4):890–904. doi: 10.1007/s00248-018-1269-y (PMC6478632; doi:10.1007/s00248-018-1269-y)
Supplement: Supplementary file 2 — Tick abundance (mean no. of ticks per 100 m2 ± SE) in Kampinoski National Park (KNP) and Mazurian Landscape Park (MLP) (DOCX 14 kb) [file 248_2018_1269_MOESM2_ESM.docx]

|  | | **Kampinoski National Park** | | | | **Mazurian Landscape Park** | | | |
| --- | --- | --- | --- | --- | --- | --- | --- | --- | --- |
| **Year** | **Season** | Females | Males | Nymphs | Total | Females | Males | Nymphs | Total |
| **2012** | **1** | 2.6 ± 1.1 | 2.1 ± 1.1 | 0.1 ± 2.4 | 4.9 ± 3.9 | 2 ± 0.6 | 1.9 ± 0.6 | 6.4 ± 1.4 | 10.4 ± 2.2 |
|  | **2** | 0.3 ± 1.7 | 1.7 ± 1.8 | 2.3 ± 3.9 | 4.3 ± 6.3 | 0.2 ± 1.2 | 0 ± 1.3 | 4.7 ± 2.8 | 4.8 ± 4.5 |
|  | **Total** | **1.5 ± 1** | **1.9 ± 1.1** | **1.2 ± 2.3** | **4.6 ± 3.7** | **1.1 ± 0.7** | **1 ± 0.7** | **5.6 ± 1.6** | **7.6 ± 2.5** |
| **2013** | **1** | 0.3 ± 1 | 0.6 ± 1.1 | 1.3 ± 2.3 | 2.1 ± 3.6 | 1.1 ± 1 | 1.6 ± 1.1 | 5.2 ± 2.3 | 7.8 ± 3.6 |
|  | **2** | 0.1 ± 1.5 | 0 ± 1.6 | 1 ± 3.4 | 1.1 ± 5.5 | 1.3 ± 1.5 | 0.1 ± 1.6 | 2.3 ± 3.4 | 3.6 ± 5.5 |
|  | **Total** | **0.2 ± 0.9** | **0.3 ± 1** | **1.1 ± 2.1** | **1.6 ± 3.3** | **1.2 ± 0.9** | **0.8 ± 1** | **3.7 ± 2.1** | **5.7 ± 3.3** |
| **2014** | **1** | 1.3 ± 1.2 | 1.8 ± 1.3 | 0.4 ± 2.8 | 3.4 ± 4.5 | 1.7 ± 0.8 | 1.1 ± 0.8 | 3.5 ± 1.8 | 6.4 ± 2.8 |
|  | **2** | 0.4 ± 1.5 | 0.9 ± 1.6 | 1.1 ± 3.4 | 2.4 ± 5.5 | nd | nd | nd | nd |
|  | **Total** | **0.8 ± 1** | **1.3 ± 1** | **0.8 ± 2.2** | **2.9 ± 3.5** | **1.7 ± 0.8** | **1.1 ± 0.8** | **3.5 ± 1.8** | **6.4 ± 2.8** |
| **2015** | **1** | nd | nd | nd | nd | nd | nd | nd | nd |
|  | **2** | nd | nd | nd | nd | nd | nd | nd | nd |
|  | **Total** | **nd** | **nd** | **nd** | **nd** | **nd** | **nd** | **nd** | **nd** |
| **Total** | **1** | 1.4 ± 0.6 | 1.5 ± 0.7 | 0.6 ± 1.4 | 3.5 ± 2.3 | 1.6 ± 0.5 | 1.5 ± 0.5 | 5 ± 1.1 | 8.2 ± 1.7 |
|  | **2** | 0.3 ± 0.9 | 0.8 ± 1 | 1.5 ± 2.1 | 2.6 ± 3.3 | 0.7 ± 1 | 0.1 ± 1 | 3.5 ± 2.2 | 4.2 ± 3.5 |
|  | **Total** | **0.8 ± 0.6** | **1.2 ± 0.6** | **1 ± 1.3** | **3 ± 2** | **1.3 ± 0.5** | **0.9 ± 0.5** | **4.4 ± 1.1** | **6.6 ± 1.7** |

**Supplementary File 2** Supplementary Table 1. Tick abundance (mean no. of ticks per 100 m^2^ ± SE) in Kampinoski National Park (KNP) and Mazurian Landscape Park (MLP)

Abbreviations: *nd* no data (no sampling performed)

Differences in tick densities (arithmetic means) were evaluated by ANOVA using models with normal errors.
