# Supplementary material for: Rickettsiales Occurrence and Co-occurrence in Ixodes ricinus Ticks in Natural and Urban Areas
Source: Microb Ecol. 2018 Oct 16;77(4):890–904. doi: 10.1007/s00248-018-1269-y (PMC6478632; doi:10.1007/s00248-018-1269-y)
Supplement: Supplementary file 3 — Prevalence of Rickettsiales in total ticks in natural and urban sites (2012–2015 average). (DOCX 15 kb) [file 248_2018_1269_MOESM3_ESM.docx]

**Supplementary File 3** Supplementary Figure 2. Prevalence of Rickettsiales in total ticks in natural and urban sites (2012-2015 average).

Abbreviations: *BNP* Białowieża National Park, *KNP* Kampinoski National Park, *MLP* Mazurski Landscape Park, *WBF* Warsaw – Bielański Forest, *WKF* Warsaw – Kabacki Forest, *WLP* – Warsaw – Łazienki Królewskie Park, *BNW* Białowieża – North-West, *BSW* Białowieża – South-West, *Ap* *Anaplasma phagocytophilum, CNM* ‘*Candidatus* Neoehrlichia mikurensis’, *Rs* *Rickettsia* spp.
