# Supplementary material for: Rickettsiales Occurrence and Co-occurrence in Ixodes ricinus Ticks in Natural and Urban Areas
Source: Microb Ecol. 2018 Oct 16;77(4):890–904. doi: 10.1007/s00248-018-1269-y (PMC6478632; doi:10.1007/s00248-018-1269-y)
Supplement: Supplementary file 4 — Anaplasma phagocytophilum infection prevalence in total I. ricinus ticks in two Subtypes of areas in two seasons (2012–2015 average) (DOCX 22 kb) [file 248_2018_1269_MOESM4_ESM.docx]

**Supplementary File 4.** Supplementary Figure 3. *Anaplasma phagocytophilum* infection prevalence in total *I. ricinus* ticks in two subtypes of areas in two seasons (2012-2015 average)

*

*p = 0.040*

Asterisk (*) marks the significant difference in one set of data (Fisher test value is given below the chart)
