# Supplementary material for: Rickettsiales Occurrence and Co-occurrence in Ixodes ricinus Ticks in Natural and Urban Areas
Source: Microb Ecol. 2018 Oct 16;77(4):890–904. doi: 10.1007/s00248-018-1269-y (PMC6478632; doi:10.1007/s00248-018-1269-y)
Supplement: Supplementary file 5 — Rickettsiales co-infection prevalence in adult I. ricinus ticks urban and natural areas (DOCX 14 kb) [file 248_2018_1269_MOESM5_ESM.docx]

| Rickettsiales co-infections in adult ticks (natural and urban areas) | | | | | | |
| --- | --- | --- | --- | --- | --- | --- |
| Year | Season | *N* | Total Rickettsiales % | Ap and Rs % | Ap and CNM % | Rs and CNM % |
| 2012 | 1 | 334 | 0.3 | 0.0 | 0.0 | 0.3 |
|  | 2 | 44 | 0.0 | 0.0 | 0.0 | 0.0 |
| 2013 | 1 | 442 | 1.4 | 0.7 | 0.7 | 0.0 |
|  | 2 | 83 | 8.4 | 4.8 | 2.4 | 1.2 |
| 2014 | 1 | 617 | 1.8 | 0.8 | 0.8 | 0.2 |
|  | 2 | 186 | 0.5 | 0.0 | 0.5 | 0.0 |
| 2015 | 1 | 237 | 3.0 | 0.0 | 2.5 | 0.4 |
|  | 2 | 87 | 0.0 | 0.0 | 0.0 | 0.0 |

**Supplementary File 5.** Supplementary Table 2. Rickettsiales co-infection prevalence in adult *I. ricinu*s ticks urban and natural areas

Red coloured – statistically significant prevalence values of effect interaction of *Year* and *Season*

1 – spring-summer, 2 – summer-autumn

Abbreviations: *N* – number of specimens, Ap – *Anaplasma phagocytophilum*, Rs – *Rickettsia* spp., CNM – ‘*Candidatus* Neoehrlichia mikurensis’
